# Enrichment experiment of ventilation air methane (0.5%) by the mechanical tower

**DOI:** 10.1038/s41598-020-63698-x

**Published:** 2020-04-29

**Authors:** Wen Wang, Jiandong Ren, Xiaojun Li, Huaibin Li, Dongyin Li, Huamin Li, Yun Song

**Affiliations:** 1 School of Energy Science Engineering, Henan Polytechnic University, Jiaozuo, 454003 China; 2 Innovation Center of Coal Work Safety, Henan Province Jiaozuo, 454003 China; 3 School of Resources and Civil Engineering, Northeastern University, Shenyang, 110819 China; 4 College of Computer Science and Technology, Nanjing Forestry University, 210037 Nanjing, China

**Keywords:** Energy science and technology, Engineering

## Abstract

Methane is one of the most important gases leading to the earth’s air pollution. Ventilation air methane(VAM) is an important part of the gas discharged into the atmosphere. The volume concentration of methane is generally less than 0.5% in coal mines. Recycling low concentration is facing challenges. To explore the law of low concentration methane enrichment, the enrichment tower for methane was designed and manufactured. The experiment was divided into two types - free diffusion and weak eddy enrichment, and eight kinds of low concentration gas experimental program. Under free diffusion conditions, the maximum methane concentration of the top (middle) tower is 0.64% (0.53%). In the condition of weak eddy field, the maximum methane concentration is 0.67% (0.69%) in the top (middle) tower. The effect of methane enrichment in the weak eddy field is obvious. Methane enrichment method under the eddy current field can greatly increase methane enrichment efficiency and achieve the goal of CMM (coal mine methane) power generation.

## Introduction

**Figure 1:**
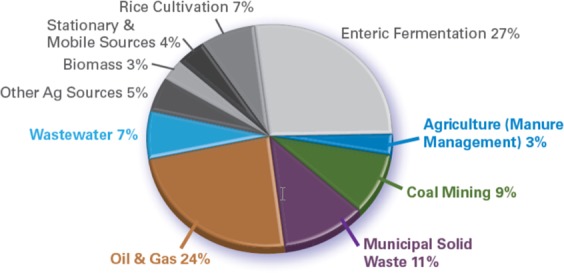
Estimated global anthropogenic methane emissions by source, 2020.

By 2020, global anthropogenic methane emissions are estimated at 9.39 billion metric tons of carbon dioxide equivalent^1^. Approximately 54% of these emissions come from the five sources targeted by the Global Methane Initiative (GMI): coal mines, oil and natural methane systems, agriculture (manure management), municipal solid waste (MSW) and wastewater (Fig. 1). Compared with the past 20 years, the rate of increase in methane concentration has increased significantly. The methane concentration rises faster than at any time in the past 20. Since 2014, are approaching the most greenhouse-gas-intensive scenarios^2^.

The methane emitted by coal mining is called “ventilation mode”, and the concentration is as low as 1% or less^3^, which is mainly divided into three parts: (**a**) mine ventilation air (0.1–1.5% CH_4_)^4^, (**b**) methane drained from the seam before mining(60–95% CH_4_)^5^, and (**c**) methane drained from worked areas of the mine(30–95% CH_4_)^6,7^. As a greenhouse gas, methane will destroy the ozone layer and increases environmental degradation, but most of it will serve as a clean resource, which can provide a lot of energy and bring huge economic benefits^8–10^. Methane emissions from coal mines have generated a large number of economic benefits in China. By the end of 2015, gas utilization reached 8.6 × 10^9^ m^3^, annual power generation reached 2.61 × 10^9^ kW h, and discharged into the atmosphere 9.4 × 10^9^ m^3^ ^11^. India is the largest coal producer, accounting for about 12% of its total production from underground mines with a maximum depth of 600 m^12^. A large amount of methane from coal mines is discharged into the air in Poland^13^.

Methane recovery and concentration increase in coal mine have major challenges. Currently, there are many methods, such as vacuum pressure swing adsorption, solvent adsorption, hydrate crystallization, and membrane separation. Particularly many works of the methane-carbon dioxide separation have been published^14,15^. When a ventilation shaft emits VAM at a concentration of about 0.4~0.5 vol% and next, the released methane is combusted and the heat is recovered by thermal flow reversal reactor (TFRR)^16^. When TFRR takes 36 hours to complete its preheating process at low concentrations (≤8%), approximately 31.61% to 46.82% of the energy can be recovered and generated^17^. Globally, by using oxidation methods, the effects of various climate change caused by ventilation in underground coal mines can be reduced by approximately 95%^18^. At the same time, coal mining emissions can be reduced to 67%^19^.

Methane separation from VAM was often used by hydrate crystallization^20^. For larger scale natural methane/nitrogen production, the option available is cryogenic distillation. The nitrogen (>96%) outlet stream purity is specified for venting, while the methane rich stream (>98%) is proper for recycling to the cryogenic process^21^. The anionic surfactant sodium dodecyl sulfate (SDS) is a promoter for the enhancement of gas hydrate formation. The methane recovery obtained under the low driving force (1.5 MPa) was approximately 33.1%^22^. The hydrate-based method is promising for separating methane from MVA. It was found that in the presence of 37.1 wt%(tetrabutylphosphonium bromide)TBPB, CH_4_ was preferentially incorporated into the hydrate phase, and the enrichment was approximately 3.5-fold^23^.

Membranes for methane separation is used by a CHA-type zeolite(Si-CHA)^24,25^. The advantage of membranes having a large pore volume,including silicoaluminophosphate, pure silica, etc.,are their ability to demonstrate high gas permeability. Membrane separation has a higher density, but the separation speed is slower and the investment is expensive.

Although the above methane enrichment method has a relatively high concentration, the gas accumulation rate is slow and requires a large amount of time. Usually, the return air volume of a coal mine is greater than 1500 m^3^/min. The above method is not suitable for recycling VAM. In 2011, the Global Methane Initiative currently consists of 38 partner countries, with government and private sector entities involved, bringing together the technology and market expertise, financing and technology necessary for international methane capture and utilization^26^.

In this study, our study is the first to study low-concentration methane enrichment experiments using eddy current fields and free-diffusion conditions. The methane concentration tower was designed,developed and then tested. The injected methane was in an isolated and non-isolated state, the results were discussed. The conclusions of different states of low concentration methane enrichment were reviewed. The effects of global warming can be controlled by reducing the concentration of methane in the atmosphere.

## Development of the Methane Enrichment Tower

### Composition of the tower

**Figure 2:**
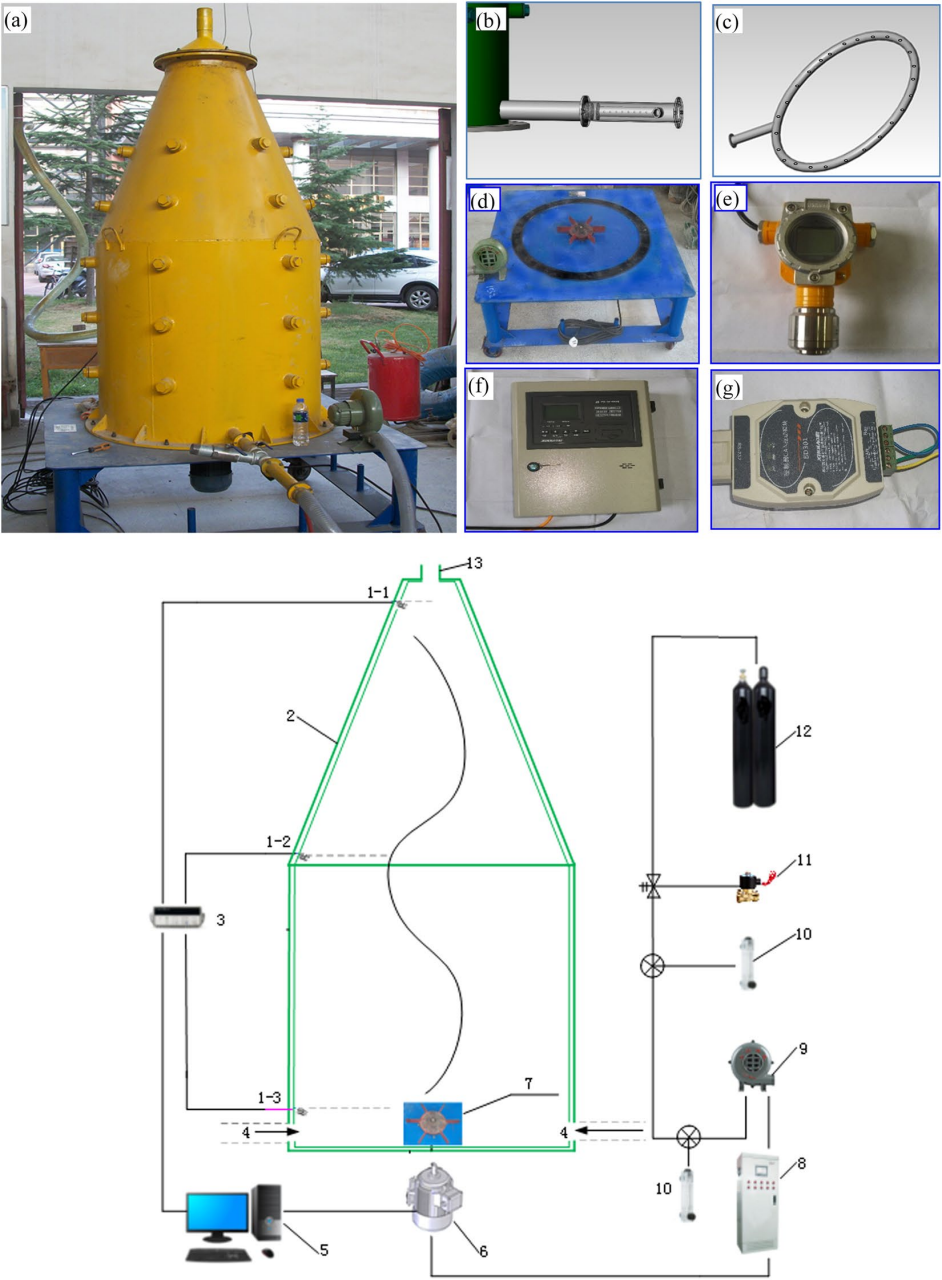
Schematic diagram of enrichment tower26. (**a**) Enrichment tower. (**b**) Three+-dimensional diagram of the mixing valve. (**c**) Porous methane inlet ring. (**d**) Base frame and blade of vortex tower. (**e**) XP3000 infrared detector. (**f**) JB-TB-AT220D control equipment. (**g**) Isolated RS485 adapter. (**h**) Structure chart of enrichment tower. 1(1-1,1-2,1-3)—methane sensor, 2—tower equipment, 3—data collection system, 4— air inlet,5—computer, 6— explosion-proof motors by a frequency conversion function, 7—blades, 8—electric control box, 9—fan, 10—flowmeter, 11—control valve, 12— methane cylinders, 13— air outlet.

The methane mechanical tower is designed and developed according to the basic principle of methane buoyancy^27^. The lower section of the device is cylindrical^27^. The upper section is conical and narrows gradually^27^. The height and diameter of the bottom cylinder are both 1 m^27^. The height of conical round table is 1m, and the material is stainless steel^27^. The tower is composed of a cylinder, explosion-proof frequency conversion motor, blade, methane and air mixing tube, methane cylinders, methane sensor, flow meter, electric control box, decompression control valve, data integrator, and computer etc^27^. The sketch and physical diagrams of the tower are shown in Fig. 2.

The operation of the tower is described as follows^27^. The methane cylinders is connected with control valve in order to adjust the output pressure and the rate of flow^27^. The methane flow is measured by a flowmeter, which is connected with the fan, and the amount of air entering the system is controllable^27^. The methane and the air flow together at a tee valve (Fig. 2b). The exterior combined tee valve is connected with the lower air inlet, which can be turned on or off^27^. An explosion-proof variable-frequency motor is installed at the bottom of the enrichment tower to drive the rotation of the inner blades^27^. The diameter and the height of the blades are 40 cm and 5 cm respectively, and the rotation rate is controlled by an electric control box to make the inner gas form vortex field^27^. Three infrared methane sensors (Model: XP3000) are installed at the top (sensor 1), middle (sensor 2), and bottom (sensor 3) of the enrichment tower, with a spacing of 1 m (Fig. 2e)^27^. Each sensor is connected with computer through a controller (Model: JB-TB-AT220D), isolated adapter (Model: RS485), graphical monitoring system transforming the methane concentration information into digital signal, and the concentration is constantly shown on the computer^27^. The flow rate of methane and air can also be constantly monitored by the computer^27^. All the test results can be automatically recorded by computers^27^.

### Experiment programs of methane enrichment

To investigate the low concentration methane (0.5 vol%) enrichment in the tower, the methane mixture is continuously divided into the free diffusion condition and the weak eddy condition, abbreviated ‘D’ and ‘E’ respectively. The free diffusion means that the methane mixture move freely under the gravity. The weak eddy conditions means that vortex field is formed by the rotation of blazes, and the methane mixture move in the vortex field. Every conditions are divided into isolated system and non-isolated system separately according to the amount of methane injected, abbreviated ‘I’ and ‘NI’ respectively. In the isolated system test, the methane is injected into the system continuously for 6 min, and then the inlet and upper outlet will be closed. In the non-isolated system test, the methane mixture is continuously injected into enrichment tower for 40 min to stabilize the concentration of methane, and the upper outlet will be kept open. The criterion for the end of the test is that the value of the methane sensor remains unchanged for 10 minutes, and the experiment is considered to be finished. When the methane and air are injected into the enrichment tower separately without a complete mixing, the molecule of methane and air do not reach the even distribution condition at this moment. The methane is called segregation status methane, abbreviated ‘S’. Since the full mixing of the air and the methane, the molecule of the methane and the air attract each other and reach an even distribution condition. At this moment, the methane is called non-segregation status methane, abbreviated ‘NS’.

**Figure 3:**
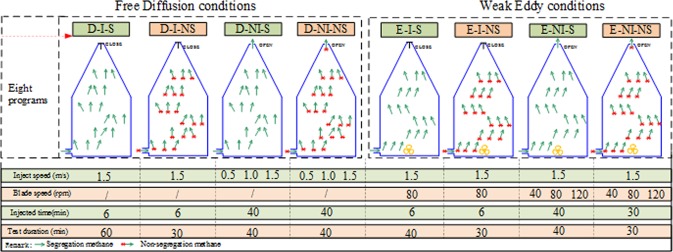
Schematics and parameters of experiments in low concentration methane.

The low concentration methane tests include eight test schemes (Fig. 3): free diffusion conditions-isolated system- segregation status methane test (D-I-S), free diffusion conditions-isolated system-non-segregation status methane test (D-I-NS), free diffusion conditions-non-isolated system-segregation status methane test (D-NI-S), free diffusion conditions-non-isolated system-non-segregation status methane test (D-NI-NS).

Weak eddy conditions-isolated system-segregation status methane (E-I-S), weak eddy conditions-isolated system- non-segregation status methane (E-I-NS), weak eddy conditions-non-isolated system-segregation status methane (E-NI-S), weak eddy conditions-non-isolated system- non-segregation status methane (E-NI-NS).

## Results of Experimental Tests

### Results of experiments under free diffusion condition (D)

1. Result of experiments under isolated system (D-I)
  Under the condition of D-I-S, methane was injected into the tower in an isolated state, the methane concentration increased rapidly. In the first 7 minutes, the concentration of methane at the bottom of the concentration tower reached 0.62% (volume concentration,) and then decreased to 0.12% (Fig. 4a). At the 4th minute, the methane concentration at the top and middle of the concentration tower did not change significantly, then the concentration increased continuously to reach a steady state. The concentration of methane on sensor 2 (middle part) is relatively stable at 30 minutes, the stable concentration of methane on sensor 1 (top) is relatively stable at 45 minutes, the concentrations are 0.54% and 0.58% respectively. The time to reach a stable methane concentration on sensors 1 and 2 increases with the increase of the tower height, the change law of the two with time is similar and has a linear relationship^27^.

**Figure 4:**
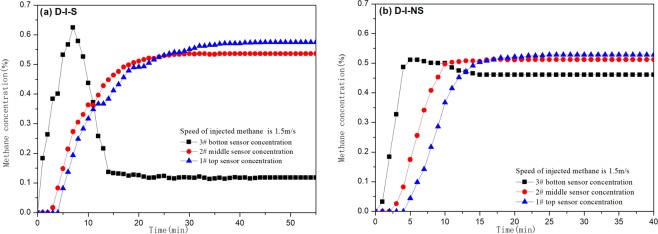
Methane concentration curves of experiments in D-I-S and D-I-NS tests.
  Under D-I-NS conditions, the non-separated methane is injected into the tower, the methane concentration on sensor 3 reaches a maximum value of 0.51%, after which a small decrease occurs, and after reaching stability, the methane concentration is 0.46% (Fig. 4b). In the middle and top of the concentration tower in the first 4 minutes, the concentration of methane did not change significantly, then the concentration continued to increase to reach a steady state. The methane concentration on sensor 1 reached stability at 25 minutes, the stabilized methane concentrations were 0.51% and 0.53% respectively. At the same time we found, the methane concentration on Sensor 3 is relatively low, reducing the amplitude is smaller.
2. Result of experiments under non-isolated system (D-NI)

**Figure 5:**
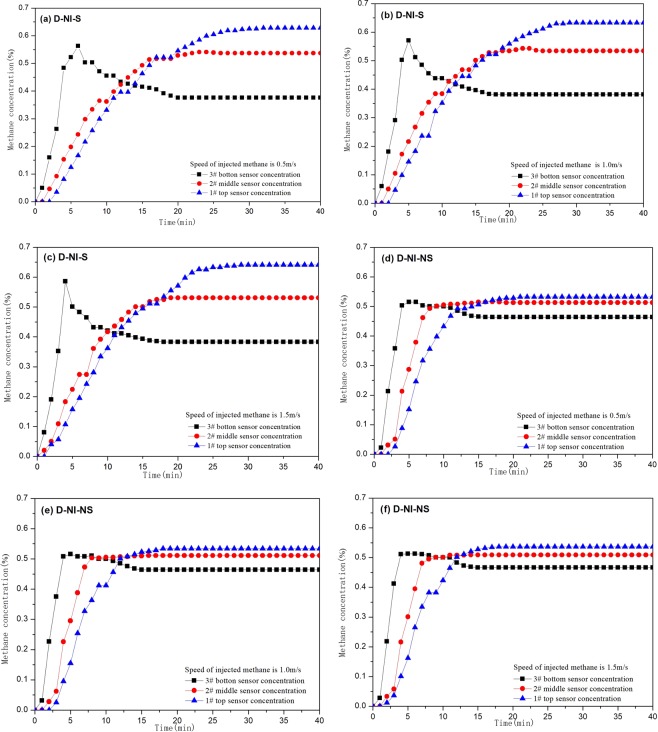
Methane concentration curves of experiments in D-NI-S tests and D-NI-NS tests.

Under the condition of D-NI-S, the methane mixture in the separated state is injected into the concentration tower, where Fig. 5 is the change law of M concentration with time and different injection speed (methane). When the injection speed is 0.5 m/s, the methane concentration (sensor 3) increased rapidly, reached a maximum value of 0.56% at 6 minutes, then gradually decreased, reached a stable value of 0.38% at 20 minutes (Fig. 5a). Sensors 2 and 1 have similar changes in methane concentration. In 3 minutes after injection of separated methane mixture, the concentration change was relatively small, and then gradually increased to a steady state. At the 25th minute, the methane concentration on sensor 2 rose to 0.54% and reached a stable value. At 32 minutes, the methane concentration on sensor 1 rose to 0.63% and reached a stable value.

Under the D-NI-S condition, when the injection speed is 1.0 m/s, the methane concentration on sensor 3 reaches a maximum value of 0.57% at the 5th minute, then gradually decreases, reaching a stable value of 0.38% at the 18th minute. (Fig. 5b). The methane concentration on sensors 2 and 1 increased less within the first 5 minutes, then increased continuously to a stable value. At the 25th minute, the concentration of the sensor 2 reached a stable value of 0.53%; at the 30th minute, the concentration of the sensor 1 reached a stable value of 0.63%. When the injection speed is 1.5 m/s, the methane concentration on sensor 3 reaches a maximum value of 0.59% at the 4th minute, then gradually decreases, reaching a stable value of 0.38% at the 18th minute (Fig. 5c); the sensor 2 and The trend of methane concentration in 1 is similar. At 19 minutes, the methane concentration on sensors 2 increased to a stable value of 0.53%; at the 30th minute, the methane concentration on sensors 1 increased to a stable value of 0.64%. The average stable concentration on sensors 1, 2, and 3 at three injection rates were 0.63%, 0.53%, and 0.38%, respectively.

Under the condition of D-NI-NS, when the injection speed is 0.5 m/s, the methane concentration (Sensor 3) reaches a maximum value of 0.52% in the fifth minute (Fig. 5d), then gradually decreases, the concentration decreases to 0.46% at 15 min. For first 3 minutes methane concentration on sensors 1 and 2 were 0% (Fig. 5d), then at 40 min gradually increased to a stable value. At 13 minutes, the concentration on sensors 2 reached a stable value of 0.51%; at 21 minutes, the concentration on sensors 1 reached a stable value of 0.53%. When the injection speed is 1.0 m/s, the methane concentration on sensor 3 starts to increase gradually, reaches a maximum value of 0.52% at 5 minutes, then gradually decreases, reaching a stable value of 0.46% at 15 minutes (Fig. 5e).The methane concentration on sensors 2 and 1 began to decrease at 3 minutes, then continued to increase to a stable value. The methane concentration on sensors 2 reached a stable value of 0.51% at 12 minutes, the methane concentration on sensors 1 reached a stable value of 0.53% at 18 minutes.

When the injection speed is 1.5 m/s under D-NI-NS conditions, the methane concentration on sensor 3 rapidly increases to 0.51% at 5 minutes, and then gradually decreases to reach a stable value of 0.47% (Fig. 5f). the concentration on sensors 2 and 1 increases small at 4 min, then they increase continuously to a stable value. The methane concentration on sensors 2 and 1 reached stable values of 0.51% and 0.54% at 12 and 16 minutes, respectively. The stable average methane concentration under the three velocities in the sensor 1, 2, and 3 are 0.53%, 0.51% and 0.46% respectively.

### Results of experiments under weak eddy condition (E)

1. Results of experiments under isolated system (E-I)
  In E-I-S condition, the segregation status methane is injected into the tower, the methane concentration on sensor 3 increased quickly, reaching maximum value of 0.55% at 7 min, and then it gradually decreases, dropping to a stable value of 0.16% at 22 min (Fig. 6a); the variation of the methane concentration on Sensor 1 and 2 is similar. During the first 3 min, the concentration varies slightly, and then it increases to the stable status continuously. At 25 min the methane concentration on sensors 2 rises to a stable value of 0.60%, while at 26 min the methane concentration on sensors 1 increases to a stable value of 0.61%.

**Figure 6:**
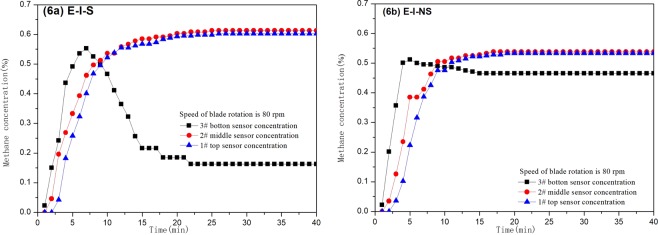
Methane concentration curves of experiments in E-I-S and E-I-NS tests.
  In E-I-NS condition, the non-segregation status methane is injected into the tower, the methane concentration on sensor 3 reaches the maximum value of 0.51% at the first 5 min, and then it begins to decrease, dropping to a stable value of 0.47% at 15 min (Fig. 6b); the variation of the methane concentration on sensors 1 and 2 is similar. During the first 3 min after injecting, the concentration varies slightly, and then it increases to the stable status continuously. At 17 min the methane concentration on sensor 2 rises to a stable value of 0.54%, while at 19 min the methane concentration on sensors 1 increases to a stable value of 0.53%. In E-I condition, the segregation status methane has obvious concentration increasing area after a short time, while there is no obvious concentration increasing area for the non-segregation methane.
2. Results of experiments under non-isolated system (E-NI)

**Figure 7:**
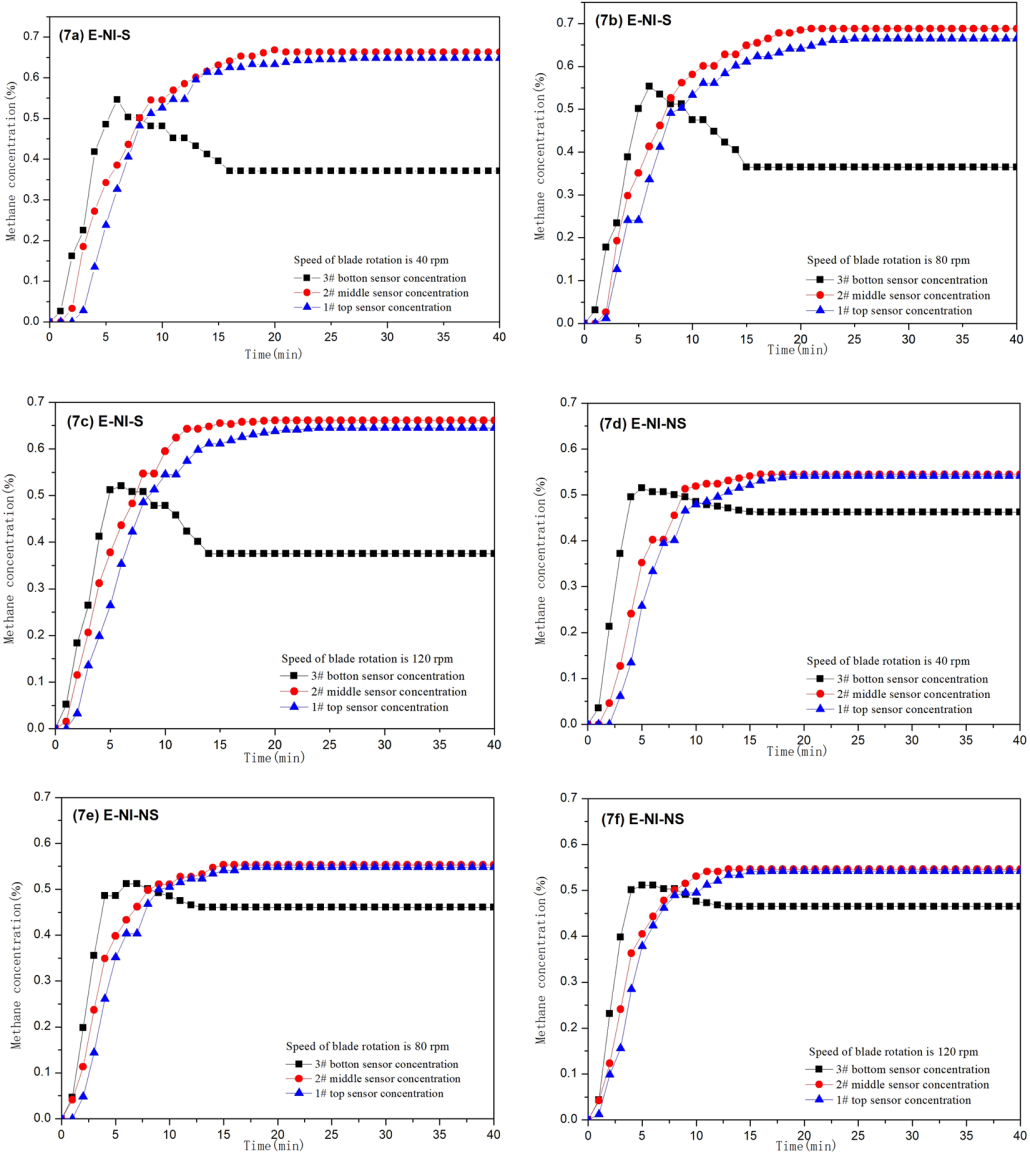
Methane concentration curves of experiments in E-NI-S and E-NI-NS tests.

In E-NI-S condition, the mixed methane with volume concentration of 0.5% is injected into the tower, and when the rotation speed of the blades is 40 rpm, only sensor 3 monitors methane at 1 min. After 3 min, all sensors monitor the methane, and after 27 min the methane concentration of all sensors tend to be stable. The methane concentration on sensors 1, 2, and 3 are 0.65%, 0.66% and 0.37% respectively (Fig. 7a). Monitoring data shows that the methane concentration in the upper and middle section of the tower is the same, and the concentration in the bottom is the lowest, which indicates that methane enrichment can be achieved in the low vortex methane field under the segregation status. When the speed is 80 rpm and 120 rpm, the methane concentration is similar to that of 40 rpm, but the methane concentration in the enrichment layer is different. When the speed is 80 rpm, the concentration of stable methane on Sensor 1, 2 and 3 are 0.67%, 0.69% and 0.37%, respectively. When the speed is 120 rpm, the stable methane concentration on sensors 1, 2 and 3 are 0.65%, 0.67% and 0.38%, respectively (Fig. 7b,c). Under the three speed conditions, the average stable methane concentration on sensors 1, 2 and 3 are 0.66%, 0.67% and 0.37%, respectively.

In E-NI-NS condition, the mixed methane with volume concentration of 0.5% is injected in the tower, the methane concentration on sensor 3 increases quickly when the speed is 40 rpm, reaching a maximum value of 0.52% at 5 min, and then it decreases gradually, dropping to a stable value of 0.46% at 16 min (Fig. 7d); the methane concentration on Sensor 1 and 2 is similar. During the first 3 min after injected, the concentration varies slightly, and then it increases to the stable status continuously. At 16 min the methane concentration on sensors 2 rises to a stable value of 0.55%, while at 19 min the methane concentration on sensors 1 increases to 0.54%, and it tends to a stable status. When the speed is 80 rpm and 120 rpm, the variation of methane concentration is similar with the methane under the speed of 40 rpm. When the speed is 80 rpm, the stable methane concentration on sensors 1, 2 and 3 are 0.55%, 0.55% and 0.47%, respectively (Fig. 7e,f). Under the three speed conditions, the methane concentration on sensors 1, 2, and 3 are 0.54%, 0.55% and 0.46%, respectively. The methane variation trend in sensors 3 is different with that on sensors 1 and 2. The methane concentration on sensor 3 increase rapidly at the beginning, reaching a maximum value at 6 min, and then it decreases gradually, reaching a stable value at 16 min, indicating that the methane concentration at the bottom increase first and then decrease, and the methane concentration at the middle and the top of the tower increase gradually until the stable status.

In E-NI condition, the time of methane concentration reaching stable in different speed rate is counted. The stable time of methane concentration on sensors 1 is 17–19 min. The time of methane concentration on sensors 2 reaching the stable state is 14–16 min. The stable time of methane concentration on sensors 3 is 12.5–16 min. It is known from data that the methane concentration at the bottom section stabilize firstly, and it stabilize from bottom to top. From the analysis to the comparison of the methane stable time between segregation and non-segregation status methane, the methane stabilization time of segregation status methane in the middle and top of the tower is longer than that of non-segregation status, while the stabilization time for segregation and non-segregation methane in the bottom of the tower is similar.

## Discussion and Analysis of Ventilation Air Methane’s Enrichment Experiment

### Discussion of the stable concentration methane

**Figure 8:**
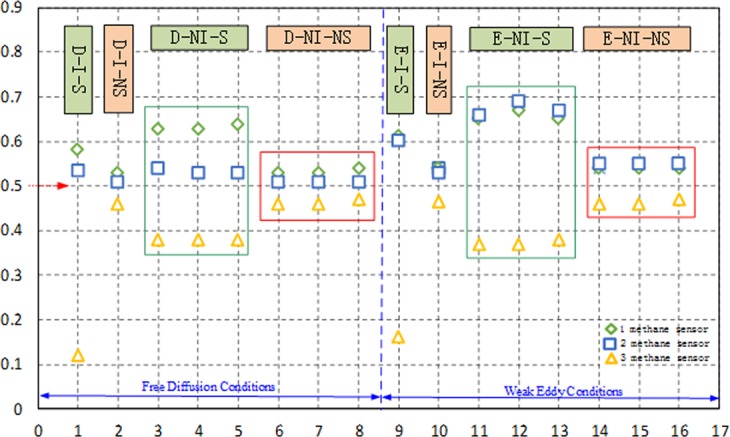
Stable concentration of methane in eight programs.

In D-I condition, the concentration of stable segregation status methane in the top of the tower is higher than that in the bottom, and the difference is 4.8 times. The non-segregation methane concentration after stability is basically equal at the upper and middle sections of the tower, but higher than that in the bottom, and the increase ratio is 10.87–15.22%, as shown in Fig. 8. In D-NI condition, the concentration of stabilized segregation methane in the upper section of the tower is higher than that in the bottom section, increasing by 18.87%; the methane concentration in the middle section is higher than that in the bottom, increasing by 39.74%, the methane concentration in the top increases 65.79% relative to that in the bottom; after the stabilization, the concentration of non-segregation status methane in the top is higher than that in the bottom, increasing 3.92%; the methane concentration in the middle is higher than that in the bottom, increasing 10.87%; the methane concentration in the top increases 15.22% relative to that in the bottom.

In E-I condition, the concentration of stable segregation status methane in the top of the tower is higher than that in the bottom, and the difference is 3.8 times. The non-segregation methane concentration after stability is basically equal at the upper and middle sections of the tower, but higher than that in the bottom, and the increase ratio is 12.77–14.89%. In E-NI condition, the concentration of stabilized segregation methane in the middle section is slightly higher than that in the top, increasing by 2.54%; the methane concentration in the middle is higher than that in the bottom, increasing by 80.36%, the methane concentration in the top increases 75.89% relative to that in the bottom; after the stabilization, the concentration of non-segregation status methane in the middle is slightly higher than that in the bottom, increasing 1.23%; the methane concentration in the middle is higher than that in the bottom, increasing 18.71%; methane concentration in the top increase 17.27% relative to that in the bottom. The concentration of segregation status methane is the non-isolated system is different in the top, middle and bottom, while the highest concentration of methane is 0.64% in the top of the tower, and the concentration increases, but the variation is not obvious.

In the enrichment process of free diffusion condition, the difference of methane concentration in the top (middle) and the bottom of the enrichment tower is obvious, and the methane concentration in the top (middle) is relatively close (except D-NI-S). The distribution of methane concentration in weak eddy current flied is the same as in free diffusion condition. In E-NI condition, the concentration in the top (middle) is higher than other schemes, with a highest concentration of 0.69%, and the concentration increases obviously, which indicates that weak eddy condition has a strong effect on the enrichment.

### Discussion of the stable methane time

**Figure 9:**
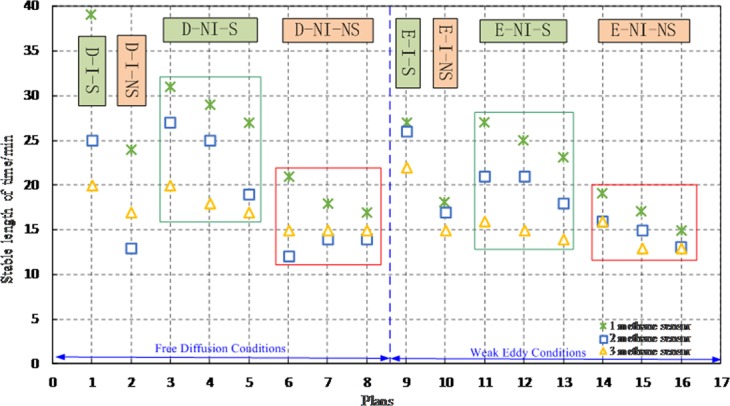
Stable time of methane in eight programs.

In D-I condition, the stabilization time of segregation status methane in the top (39 min) of the enrichment tower is longer than that in the bottom (20 min), shown in Fig. 9. The stabilization time of non-segregation methane in the top of the tower (24 min) is longer than that in the bottom (17 min), and the time in the two states was prolonged by 95% and 41.18%, respectively. In D-NI condition, the stabilization time of segregation status methane in the top (29 min) of the enrichment tower is longer than that in the bottom (18.3 min); the stabilization time of non-segregation status methane in the top (18.6 min) of the enrichment tower is longer than that in the bottom (15 min), and the time in the two states was prolonged by 58.47% and 24%, respectively. Under the free diffusion condition, the stabilization time of segregation methane in the top, middle, and bottom in non-isolated system are the shortest.

In E-I conditions, the stabilization time of segregation status methane in the top (27 min) of the enrichment tower is longer than that in the bottom (22 min). The stabilization time of non-segregation methane in the top of the tower (18 min) is longer than that in the bottom (15 min), and the time in the two states was prolonged by 22.73% and 20%, respectively. In E-NI condition, the stabilization time of segregation status methane in the top (25 min) of the enrichment tower is longer than that in the bottom (15 min); the stabilization time of non-segregation status methane in the top (17 min) of the enrichment tower is longer than that in the bottom (14 min), and the time in the two status was prolonged by 66.67% and 21.43%, respectively. Under the weak eddy condition, the stabilization time of segregation methane in the top, middle, and bottom in non-isolated system are the shortest.

It known from the enrichment time in eight schemes, E-NI-NS scheme requires the shortest time, which indicates that weak eddy condition has a strong effect on enrichment.

### Theoretical analysis of enrichment methane

1. Analysis of Free diffusion condition
  Due to buoyancy, the low-density methane rises relative to the air. For the static distribution, no temporal variations are considered. Due to buoyancy forces, the methane concentration increases approximately 0.10% for every 10-m rise in vertical height, and the increase in methane density is small. The magnitude of methane enrichment necessary for industrial applications is thus not possible by only concentrating methane at increased heights.
2. Analysis of Eddy current flied condition

When methane and air are conducted by eddy current flied, the methane rising spirally appears in a turbulent mixing state. In the mixing process, the inertial force of the vortex field needs to overcome the buoyancy and the viscous shear stress, and the Richardson number is introduced, which is the ratio of buoyancy and inertial force. The ratio of the magnitude indicates the degree of methane and air mixed. Using turbulence theory, the improved Richardson number(R) is derived^16^:

1 $$R=\frac{({\rho }_{1}-\overline{\rho (\xi )})g\,\cos \,\theta }{{\rho }_{1}\bar{V}(du/dy)}$$

where *ρ*_1_ is the density of air, kg/m^3^; *ρ*_2_is the density of methane, kg/m^3^; *θ* is the spiral angle of methane and air; *V*_1_ is the average speed of air, m/s; *V*_2_ is the average speed of methane layer, m/s; $\bar{V}$ is the average speed of methane and air; $\overline{\rho (\xi )}$ is the average mixed density of methane and air, kg/m^3^.

Because the volume concentration of injected methane reaches 0.50%, methane layer(h) appears relatively thin, thus *h* ≤ 0.2*r*,where r is the diameter of the tower. According to Prandtl mixing-length (l), thus *l* = *ch*, where c is the Carmen constant, approximate 0.4. Formula (2) can be converted to:

2 $$R=\frac{({\rho }_{1}-{\rho }_{2})chg\,\cos \,\theta }{{\rho }_{1}({V}_{1}^{2}-{V}_{2}^{2})}=K\frac{h\,\cos \,\theta }{{V}_{1}^{2}-{V}_{2}^{2}}$$

where K is the constant, $K=\frac{({\rho }_{1}-{\rho }_{2})cg}{{\rho }_{1}}$;

Under segregation methane condition, due to the difference in methane and air (the difference in molecular mass), both methane and air rise spirally. With the inertia force, the difference between V1 and V2 is getting large and R number gets relatively small. It shows that the methane molecules are less buoyant and the inertial force appears more obvious or plays a leading role. Under the effect of shearing on methane in vortex flow field, methane and air showed layered enrichment and methane concentration increased. Because the upper part of the vortex tower is tapered, the methane velocity at the top vortex field is enhanced, and the methane concentration is more easily increased at the top of the vortex tower.

Under non - segregation methane condition, the molecules of methane and air are attracted to each other and V1 and V2 shows nearly the same. Theoretically, R number is close to infinity, the mixed methane and air have large potential energy, and the potential energy plays a leading role while the kinetic energy is less pronounced. The vertical uplift characteristics of the mixed methane and air play a dominant role, and the vortex rotation does not rise significantly. Because the uplift feature requires strong kinetic energy to separate the mixed methane and air, it is difficult to achieve in the weak eddy current flow field. Therefore, the methane concentration enrichment in the non-segregation state is not obvious.

The experimental results show that the concentration of the segregation methane increases significantly under the condition of weak eddy current flow, and the concentration increases from 0.50% to 0.70%. Under the condition of increasing eddy current field strength, methane concentration enrichment will be more significant. The use of vortex towers enriches methane gradually, increasing the concentration step by step, and further reaching the industrial methane concentration.

## Conclusions

The methane concentration tower was designed and manufactured, and the monitoring data was automatically stored by monitoring the change in concentration in real time. Eight methane enrichment experiments were carried out according to the design of the scheme. The experimental results show that: (a) under D-NI-S and free diffusion conditions, methane enrichment is the best, with concentrations in the lower, middle and top of the methane concentration tower. The order of 0.38%, 0.53% and 0.64% is in turn; (b) E-NI-S methane enrichment is better under weak vortex conditions, and weak eddy current plays an important role in methane concentration enrichment. The methane concentrations at the top and the top of the tower are 0.37%, 0.69%, and 0.67%, respectively; (c) under the conditions of free diffusion and weak eddy current field, the methane and air are used to diffuse and enrich methane, and the enrichment concentration increases slightly; Under the condition of weak eddy current and methane floating, the enrichment effect of low concentration methane is better than free diffusion. According to the above, the low concentration of methane enrichment in the weak vortex region has a good development prospect.
